# The Chloroform and Ether Controversy

**Published:** 1892-03-12

**Authors:** 


### THE PRACTITIONER'S MIRROR AND RETROSPECT.

[*The Editor will be glad to receive offers of co-operation and contributions from members of the profession. All letters should be addressed to* THE EDITOR, THE LODGE, PORCHESTER SQUARE, LONDON, W.]

### MEDICINE.

### THE CHLOROFORM AND ETHER CONTROVERSY.

The rival merits of these two anæsthetics were the subject of bitter controversy almost from their birth. Since the second Hyderabad Commission in 1890 the number of contributions bearing on the question is truly enormous, but though many of the authors are men of eminence in the scientific world and most skilled observers, the controversy leaves off where it began. If we turn up the medical journals of nearly half a century ago, we find that much the same arguments were employed by the respective upholders of these two anæsthetics.

In opening a discussion on Anæsthetics at the Bristol Medico-Chirurgical Society last month, Dr. Henry Marshall referred to the long and careful search for a good anæsthetic by the late Sir James Simpson before he succeeded in discovering the properties of chloroform. A small quantity had been sent by a friend to Sir James, but he considered it so unpromising in appearance that it was for a long time put aside and forgotten. However, it was produced one evening when Simpson and his assistants, the late Matthews Duncan and another, had met, as was their wont, to experiment on themselves by placing the drug in tumblers, and the tumblers in warm water, and inhaling the vapours given off as they sat round the table. Simpson fell off his chair, and fortunately soon came round to find his two assistants completely anæsthetised, but not too far under for recovery. Now, Professor Syme taught that chloroform poisoning always caused respiratory failure before cardiac failure, and that all that was needed to ensure absolute safety as far as the anæsthetic was concerned was to give the chloroform with plenty of air, and to leave it off as soon as the slightest failure or embarrassment of respiration was observed, and, if necessary, do artificial respiration. He never felt the pulse or paid the least regard to the condition of the heart. So far from his regarding any heart disease as a contra-indication for chloroform, it was in itself the strongest reason for its administration when an operation was required, inasmuch as it quieted the heart, lessened shock, and greatly diminished the risk of cardiac failure reflexly from syncope. As a great many of the patients who were refused chloroform in London because they had heart disease, rendering its administration dangerous to life, went North to Syme, his practice was a severe test of the theories he held. And what was the result? Syme gave chloroform, either with his own hand or by an assistant, in 20,000 cases without a death. Dr. Marshall said that he never heard of any pupil of Syme's who ever recanted from the faith in the safety of chloroform administered with careful watching of the respiration. Surgeon-Major Lawrie, a pupil of Syme's, has given chloroform in 25,000 cases without a death. Forty-five thousand cases without a death! truly such a fact goes a long way towards proving the correctness of Syme's views. But "death under chloroform is not always death from chloroform." Dr. Marshall recalled an occurrence in the operating theatre of the Edinburgh Royal Infirmary on the morning when chloroform was to be administered there for the first time in public. No less than seven hundred onlookers were gathered together to watch and criticise the effects of this newly-discovered marvel which rendered operations painless. Syme was to give the anæsthetic, but was delayed. The first patient required but a trivial operation, and as it was considered inadvisable to give the chloroform in his absence, the slight operation was commenced without it. A trivial operation, only an abscess to be opened! But on the first entry of the knife the patient's jaw fell, and the ashy lips parted in death. He died from reflex inhibition of the heart. Now, had that patient had but a single whiff of chloroform, what, asked Dr. Marshall, would have been the effect of that death on the seven hundred eager critics? Chloroform would have been then, as it often is now, accused most unjustly of causing the death.

But if one starts with the fixed and immovable conviction that chloroform must be, and is always safe if cautiously given, and cannot kill, it is quite natural that no fatal result under chloroform is attributed under any circumstances to the anæsthetic. Does not this account for the extraordinary success of the Edinburgh school of chloroformists? It is difficult otherwise to account for the fatalities that have occurred every now and then in the practice of the most skilful and cautious anæsthetists.

The first Hyderabad Commission of 1888, appointed to investigate the action of chloroform, sustained the observations of Surgeon-Major Lawrie, and came to the conclusion that chloroform may be given by inhalation with perfect safety, and without any fear of accidental death, if only the respiration be carefully attended to throughout.

The result of the second Hyderabad Commission, on which Dr. Lander Brunton represented the *Lancet*, is based on more than four hundred experiments on dogs, goats, monkeys, and horses. The observations were made upon some of the animals in health, in some where a prolonged dieting of farinaceous or fatty food, or the administration of phosphorus, &c., had artificially induced fatty degeneration of the heart, muscle, and other tissues. The chloroform was given in every conceivable manner, and in a large number of cases strychnine, morphine, or atropine, either alone or in combination, were given hypodermically previous to the anæsthetic. Wherever the chloroform was pushed the respiration in every single instance ceased before the heart, and if artificial respiration was commenced within half a minute after respiration failed, recovery almost invariably occurred.

Chloroform was found to lower blood pressure, though when administered freely diluted with air the fall in blood pressure was extremely gradual, and this is probably due to vaso-motor dilatation from partial failure of the vaso-motor centre. Dr. Archie Stockwell* draws attention to the great importance of this fact: "We are further indebted to the Hyderabad Commission itself for more precise knowledge of another danger pertaining to chloroform, viz., the importance of the part played by vaso-motor paralysis in the causation of death. If we accept the report without reserve, that chloroform has no power of paralysing the heart, we have one danger removed, only to be replaced by another equally as great in every respect, viz., paralysis of the vaso-motor centre. This, it is admitted, may set in suddenly and without warning; is as unremediable as cardiac paralysis, and equally as fatal. 'When death occurs from vaso-motor paralysis, it is clearly imaginable that the vaso-motor centre may be hopelessly damaged, or rapidly reaching that stage, while the pulse is still present, and the heart attempting to keep up the pressure; so that for some seconds there may be a deceptive condition, in which both pulse and respiration are present, yet a fatal termination is imminent' (Alexander Wilson). That this death from vaso-motor paralysis does occasionally occur in man is shown by the very rapid running pulse mentioned in some cases as preceding fatal termination. The deduction to be drawn is obvious, and it cannot

*Therap. Gaz., Sept. 15, 1890.

be too strongly insisted upon, that the work of the Hyderabad Commission gives us no greater (but rather less) confidence in chloroform than we had before."

It must also be borne in mind that, assuming the correctness of the Hyderabad Commission's observations of facts, it is only proved that chloroform paralyses respiration before the heart in animals. The conclusion that its action is invariably the same in man is an assumption, although based on analogy. Now nothing is more misleading than to assume that the action of any drug on man is the same as its action on animals. Ostertag's experiments on the action of chloroform inhalation in producing fatty degeneration of the internal viscera were made on rabbits, guinea-pigs, rats, cats, dogs, and pigeons, and the results showed that the effects of chloroform differ very considerably in this respect in different groups of animals. Surely if the effects in producing fatty degeneration differ in groups of animals so closely allied, it is not difficult to understand that its effects on the respiratory and vaso-motor centres in man differ from those in animals. In spite of the greatest caution deaths do occur under chloroform, and in the majority of instances the pulse has been noticed to suddenly fail, while respiration continues. As this sometimes happens before any incision has been made, it is useless to urge that they are instances of reflex inhibition, and are deaths " under chloroform, but not from chloroform." The immediate inversion of the patient —Nelaton's method—has been followed by recovery in some instances, and as inversion impedes and not assists failing respiration, it seems ridiculous to insist that these cases of recovery from inversion were due to respiratory failure, which the flurried anæsthetists have overlooked, and attributed to heart failure.

We believe that positive clinical experience is worth more than any amount of negative evidence, no matter what the evidence of the physiological laboratory may be.

